# Introducing Greek Guidelines for the Diagnosis and Treatment of Adverse Health Effects of Occupational Exposure to Metals

**DOI:** 10.29024/aogh.2330

**Published:** 2018-08-31

**Authors:** Evangelia Nena, Sotirios Koupidis, Eleni Douna, Anastasia Kikemenis, Georgios Dounias

**Affiliations:** 1 Laboratory of Hygiene and Environmental Protection, Medical School, Democritus University of Thrace, Alexandroupolis, GR; 2 Department of Occupational and Industrial Hygiene, National School of Public Health, Athens, GR

## Abstract

**Background Aim:**

Implementing guidelines in the practice of occupational health is a high-priority need, since their use can reduce the variability in practice and increase professional efficiency, resulting in higher quality of health care services. The aim of this report is to provide information regarding the development process of the first series of national guidelines for the diagnosis and treatment of adverse health effects occurring after occupational exposure to hazardous metals in Greece. This task was recently initiated in Greece as part of the health care reform system in the context of the ongoing financial crisis.

**Methods:**

The following metals: arsenic (As), cadmium (Cd), chromium (Cr), lead (Pb), mercury (Hg), and nickel (Ni), which are the most commonly encountered in Greece, were selected to be studied. A systematic review of medical literature resulted in 94 review papers of the initial 3,932 eligible according to the inclusion criteria.

**Results:**

For each metal, an extensive report was produced, including physical and chemical properties, routes of exposure, health hazards, medical surveillance, occupational exposure limits, protection and control measures, first aid and rescue, and waste management. Special attention was paid to environmental exposure data, effects on children health, and necessary laboratory examinations.

**Conclusions:**

The first series of guidelines, regarding diagnosis and treatment of individuals who have been occupationally exposed to metals is available; it was recently published in Greece by the National School of Public Health, aiming at helping occupational health practitioners enhance the quality of their provided services.

## Introduction

Adverse health conditions due to metal exposure is a global health problem that is expressed by numerous and usually undifferentiated symptoms, depending on the duration, dose, method of exposure, and medical condition of the exposed individuals [1,2,3,4]. Health providers in occupational settings and in primary health care (PHC) should therefore have direct access to evidence-based knowledge, such as evidence-based practice guidelines, in order to support their medical decisions and to be able to diagnose and treat medical conditions attributed to metal exposures [5].

As in most European countries, the provision of occupational medicine services in Greece is obligatory for both public and private businesses of more than 50 employees or for smaller enterprises with employees exposed to noxious substances such as carcinogens and harmful biological agents. In all cases, costs are covered by the employer. It is estimated that approximately 24% of the employed population has free access to occupational medicine services, which is provided by 135 occupational medicine (OM) specialists and additionally 500 to 800 physicians of other specialties [6]. Not surprisingly there is quite a difference between the legal protection frameworks and the field realities of poor access and variable quality, deriving from a fragmented labor market, deleterious austerity policies in response to the economic crisis, a critical lack of hard data on the extent of the occupational health problems, shortages of qualified staff, lack of representation in the academic community, and a decades-long underinvestment in producing a technical cadre for the legally required occupational health services (OHS).

Greece is a country of micro- and small private enterprises (of respectively fewer than 10 or between 10 and 49 employees). In 2016 there were 229,361 such enterprises without the legal obligation to provide OHS to their employees. There were also 3,249 medium (50–249 employees) and 541 large (250+) private enterprises, i.e. only 1.6% of the employers, with the obligation to do so [7].

The data collection on occupational morbidity and disability has always been incomplete but this phenomenon has become more problematic during the economic crisis [8]. Occupational morbidity and disability had to be monitored by 70 different public insurance organizations, of which only the major one, which covered approximately 50% of the population, had a recording and notification system. The merge of the insurance schemes, initiated as part of the health reform system, is incomplete to date, and the situation of the monitoring of occupational morbidity and disability remains unclear.

The reporting of occupational diseases is mandatory, but the very low compensation awards represent poor incentives. Therefore, such diseases very often are not attributed to the occupation, leading to a significant under-recognition and under-registration [8]. Finally, specialization training in OM lasts four years and includes 28 months of clinical training, 12 months of academic training, and eight months of practical training [9].

OM physicians encounter a wide spectrum of pathologic conditions and disorders and to them the existence of guidelines is essential in order to reduce variability in their practice, increase their professional efficiency, and ensure their patients’ safety [10]. Therefore, scientific societies and organizations such as the American College of Occupational and Environmental Medicine in the United States [11] or the NHS in the United Kingdom [12] have developed guidelines, so as to improve employees’ health by increasing the efficiency of diagnosis and the effectiveness of the proposed treatments and to enhance in general the quality of occupational medical care in a professional and timely manner.

Unfortunately, OM practice in Greece is considered to be on the sidelines of health policy priorities and, as a result of the economic crisis, is considered to be between neglect and erosion. However, the health care reform that was initiated in Greece, which aims at investing in occupational and environmental health, led to the decision of developing a series of PHC guidelines.

The Department of Occupational and Industrial Hygiene of the National School of Public Health (NSPH) developed national guidelines for PHC professionals, so as to assist them in the recognition, diagnosis, and treatment of individuals – namely exposed employees – with health conditions caused by exposure to hazardous metals, in line with previous international guidelines [13]. A parallel goal was to aid in the recognition and monitoring of occupational diseases after metal exposure. The present study outlines the procedure that was followed in order to develop the first series of guidelines regarding diagnosis and treatment of adverse health conditions resulting from occupational exposure to toxic metals.

## Methods

The study group of the NSPH focused on the following six metals: arsenic (As), cadmium (Cd), chromium (Cr), lead (Pb), mercury (Hg), and nickel (Ni), based on the fact that five metals (arsenic, cadmium, chromium, lead, and mercury) are characterized by a high degree of toxicity and rank among the priority metals of public health significance [14]. Nickel was added to the study because its concentrations are known to be particularly high in Greece, a fact verified by the sampling conducted by the European Union authorities in 22,000 locations. The report that was produced concluded that the highest density of samples with nickel concentrations above the higher guideline value were measured in Greece [15].

The search strategy of the study group referred to selection of scientific studies with already existing guidelines referring to occupational exposure to the six metals under study, published after 2000, comprising at least an abstract in English. An additional criterion was the eligibility of the guidelines to be applied in occupational settings and/or primary health settings, the latter for the treatment of exposed individuals, with special attention to the current conditions in Greece, especially in the context of financial restrictions.

A Medical Search Heading (MeSH) search in PubMed, EMBASE, Google Scholar and websites of international and national organizations in the field of medicine, chemistry, and environmental science resulted finally in 94 review papers, out of the initial 3,932 eligible.

## Results

For each metal, an extensive report was produced, which is freely available online [16]. Each 10- to 15-page report includes the following: an introductory description of the physical and chemical properties of each metal, the routes of exposure, and all known health hazards. Moreover, the occupational settings and proceedings where exposure can occur, the occupational exposure limits, the protection and control measures, the suggested medical surveillance practices, and the optimal personal protective equipment are discussed.

Furthermore, each report displays all necessary emergency, first aid, and rescue measures for the treatment of adverse health effects. Special attention was also paid to environmental exposure data and waste management procedures. Finally, in two special chapters, all available laboratory examinations for the measurement of toxic metal levels in biological fluids, as well as handling of exposure in pediatric populations, are mentioned.

## Discussion

Health disorders due to toxic metal exposure in occupational settings are expressed by various symptoms, which can be difficult to differentiate [2,3,4]. As previously mentioned, the importance of guidelines in medical practice is well accepted among scientific medical societies, because they are essential in order to reduce variability in medical practice, increase professional efficiency, resulting in quality of health care services and assurance of patient safety [5].

Additionally, the introduction of guidelines can be a significant part of a health care reform process, which can lead to more efficient use of limited resources, a parameter that should not be neglected in all countries facing fiscal restrictions, like Greece.

The current study describes the procedure that was followed to publish the first series of national guidelines for the diagnosis and treatment of adverse health conditions after occupational exposure to six metals that are common in Greece.

Certainly there are limitations in this work; for example, it did not comprise publications in languages other than English, excluding thus a significant part of literature produced in under-development countries, where exposure is more common and probably better studied. However, guidelines’ introduction is important, and can be seen as a first step for the overall modification of the occupational health practices that are applied presently.
